# Combined Minimally Invasive Percutaneous Nephrolithotomy and Retrograde Intrarenal Surgery for Staghorn Calculi in Patients with Solitary Kidney

**DOI:** 10.1371/journal.pone.0048435

**Published:** 2012-10-30

**Authors:** Dehui Lai, Yongzhong He, Yuping Dai, Xun Li

**Affiliations:** 1 Department of Urology, The First Affiliated Hospital of Sun Yat-sen University, Guangzhou, Guangdong, China; 2 Department of Urology, The Fifth Affiliated Hospital of Guangzhou Medical University, Guangzhou, Guangdong, China; University of Louisville, United States of America

## Abstract

**Background:**

To present our experience with simultaneous combined minimally invasive percutaneous nephrolithotomy (MPCNL) and retrograde intrarenal surgery (RIRS) to manage patients with staghorn calculi in solitary kidney, and evaluate the safety, efficiency and feasibility of this approach.

**Methodology/Principal Findings:**

The study included 20 patients with staghorn calculi in solitary kidney. Demographic characteristics, stone location and surface area were recorded. After informed consent, the patients underwent one stage MPCNL firstly. Combined second stage MPCNL and RIRS simultaneously were performed at postoperative 5–7 days. Operative parameters, stone-free rate (SFR), stone analyses and complications were evaluated. Serum creatinine (Scr), glomerular filtration rate (GFR) and chronic kidney disease (CKD) were measured preoperatively, postoperatively at 1 month, and each follow-up visit. All patients had staghorn stones involving multiple calyces. The mean stone burden was 1099.9±843.95 mm^2^. All patients had only one percutaneous access tract. The mean whole operative duration was 154.37±32.45 min. The mean blood loss was 64 (12–140) ml. The final SFR was 90%. During the 1-month follow-up study period, four patients improved in CKD stage. Two patients who had CKD (stage 5) still needed dialysis postoperatively. Mean Scr of the rest patients preoperatively was 187.16±94.12 compared to 140.99±57.92 umol/L by the end of 1-month follow-up period (p = 0.019). The same findings were observed in GFR in that preoperatively it was 43.80±24.74 ml/min and by the end of the 1-month follow-up it was 49.55±21.18 ml/min (p = 0.05).

**Conclusions/Significance:**

Combined MPCNL and RIRS management effectively decrease the number and size of percutaneous access tracts, which is safe, feasible, and efficient for managing staghorn calculi in solitary kidney with satisfactory SFR and reducing blood loss, potential morbidity associated with multiple tracts. The approach did not adversely affect renal function at both short-term and long-term follow-up.

## Introduction

Staghorn calculi are branched stones that occupy the renal pelvis contain one or more caliceal extensions [1]. A heightened risk of chronic renal failure and life-threatening urosepsis are well-known risks of untreated staghorn calculi, especially for patients with solitary kidney [2].Percutaneous nephrolithotomy (PCNL) has evolved into widely accepted and primary recommended management for staghorn calculi since its first described in 1976 [3].However, The stone-free rate (SFR) of staghorn calculi following PCNL monotherapy is only 74% to 83% [4]. Furthermore, PCNL among the staghorn patients is associated with potential risk, such as significant hemorrhage, urosepsis, urine leakage, uncontrolled hemorrhage, embolization, nephrectomy, or even death [5]. In patients with solitary kidney, staghorn calculi occupying several calyces had to require multiple access tracts to achieve complete stone-free. It may be associated with greater potential risk. Performing PCNL with a small size tract (12–20 F), named minimally invasive percutaneous nephrolithotomy (MPCNL), may decrease the injured of the kidney and operative morbidity, comparing to the standard PCNL with (26–30 F) tract [6], [7], [8]. But the disadvantages of MPCNL are relatively low efficiency to fragment large stones and high intrapelvic pressure than the standard PCNL.

Retrograde intrarenal surgery (RIRS) is become popular, benefited from the advance in flexible ureteroscopic (FU) instrumentation and holmium laser lithotripsy. It allows retrograde access to the entire intrarenal collecting system in treating renal calculi to achieve complete stone-free, and it is safer than PCNL. However, RIRS is burdened with high rates of fiber breakage and lower efficiency in larger stone [9]. Less ideal for manage the whole staghorn calculi, RIRS can be used as an auxiliary procedure of MPCNL to reach peripheral renal residual calculi in patients with solitary kidney, on the purpose of stone-free and keeping the maximum renal function.

In this study, we evaluated the safety, efficiency and feasibility of combined MPCNL and RIRS on management of staghorn calculi in patients with solitary kidney and determined long-term renal functional results.

## Materials and Methods

We obtained approval for this study from the ethics committee of the fifth affiliated hospital of Guangzhou Medical College. Written informed consent was obtained for all the participants in our study. We performed a retrospective analysis of 20 patients (staghorn calculi involving multiple calyces) who underwent single-tract MPCNL and RIRS combination for staghorn calculi in a solitary kidney between July 2009 and March 2011 at the Urology Department of the 5th affiliated hospital of Guangzhou Medical College. Stones was divided into complete [7 cases, 35%, (occupying all calyces and renal pelvis, or filled >80% of the renal collecting system)] and partial [13 cases, 65%, (occupying renal pelvis, or at least 2 or more calyces)] staghorn calculi [10]. Stone burden was assessed as the surface area and calculated according to the European Association of Urology guidelines [11].The causes of solitary kidney were congenital in three cases (15%), previous contra lateral nephrectomy in 10 cases (50%), and nonperfused contra lateral in 7 cases (35%).

Patient characteristics analyzed were age, sex, BMI, causes of solitary kidney, previous renal intervention history, hypertension and diabetes. Preoperative tests, such as blood routine tests, coagulation tests, serum biochemistry, urinalysis, urine culture, ultrasonography and plain X-ray were recorded. Computerized tomography (CT) was done routinely, in order to analyze the size and location of the stone, the thickness of renal parenchyma, the anatomical structure and plan the target percutaneous tract and calyx. Preoperative antibiotics were administered to the results of urine culture.

For each patient, serum creatinine (Scr) and glomerular filtration rate (GFR) were measured before and after the whole procedure, and each follow-up visit. GFR was calculated using the Cockroft and Gault formula by Scr [12], [13]. Chronic kidney disease (CKD) was classified by the National Kidney Foundation Kidney Disease Outcome Quality Initiative (NKFK/DOQI) classification system [14]. CKD stages 1 to 5 were classified as a GFR that was normal, mildly, moderately, severely decreased and a requirement for dialysis or kidney transplantation (greater than 90, 60 to 89, 30 to 59, 15 to 29 and less than 15 ml/minute, respectively). The Scr, GFR and CKD stage during preoperative period were compared with those at the follow-up visit.

### Surgical Technique

#### One-stage single-tract MPCNL

**Figure 1:**
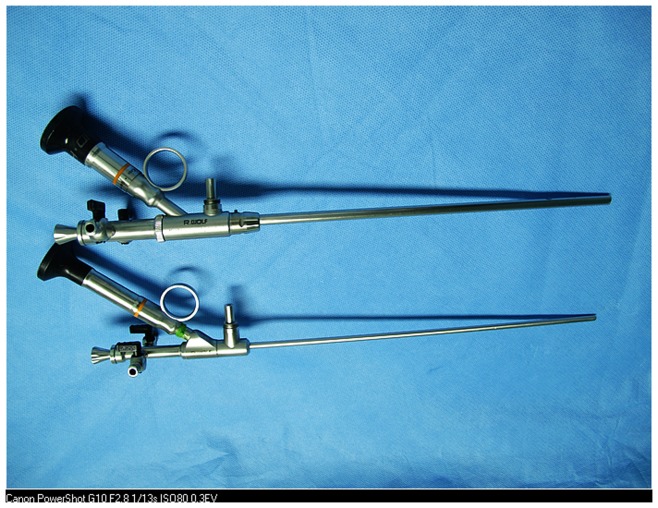
The 8.5/12 F rigid nephroscope and 18 F rigid nephroscope (designed by Li Xun, Richard Wolf, German).

All procedures were performed under continual epidural anesthesia. Patients were inserted a 5 F ureteral catheter into the target ureter and placed a 16 F Foley catheter in the bladder under lithotomy position. Then, patients were placed in the prone position with a pillow under the abdomen. MPCNL was performed under C-arm fluoroscopic guidance using an 18 gauge needle for access to the target calyx. A posterior middle calyx puncture via the 11th intercostal space between the posterior axillary line and scapula line was preferred. The needle was directed into the papilla of the intended posterior calyx using the ‘‘bull’s-eye’’ technique [15]. Once the needle was properly placed into the calyx, a 0.035-inch guidewire was inserted through the needle into the intrarenal collecting system. Tract dilatation was serially accomplished using Amplatz dilators from 8 F to 20 F.A matched diameter working sheath was advanced into the collecting system. The stones were fragmented with a holmium laser or pneumatic lithotripter through an 8.5/12 F rigid nephroscope (designed by Li Xun, Richard Wolf, German)(Figure 1), and with Cyberwand® dual probe ultrasonic intracorporeal lithotripter through 18 F rigid nephroscope (designed by Li Xun, Richard Wolf, German) (Figure 1).

The left valve of nephroscope was connected with an endoscopic pulsed perfusion pump, and the right valve was connected with Suction. The big fragments were removed with a forceps, and small fragments were pushed out with an endoscopic pulsed perfusion pump and suck out with suction. During the procedure, remove the stone in the renal pelvis and the low calyces, as for as possible. Meanwhile, rudely inserting the nephroscope to arrive the peripheral calyx is not supported. Finally, a 6 F modified Double–J stent, which had two open tails, was inserted via the percutaneous tract with the assistance of guidewire, and a matched size balloon nephrostomy tube was inserted in the involved calyx. The operative duration was calculated from the time of percutaneous puncture to the completion of nephrostomy tube placement.

On postoperative day 1 or 2, routine KUB and retrograde nephrostogram were done in all patients to assess for residual stone fragments, urinary leakage and infrarenal obstruction. On postoperative day 5∼7, second stage procedure was done for the patients.

### Combination of Single-tract MPCNL and RIRS

**Figure 2:**
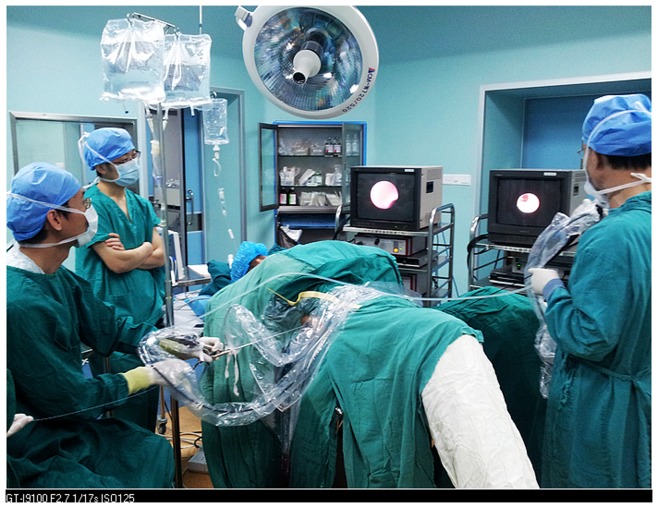
Sigle-tract minimally invasive percutaneous nephrolithotomy and retrograde intrarenal surgery were performed simultaneously in Galdakao-modified supine Valdivia (GMSV) position by two urological groups.

The procedures were performed under continual epidural anesthesia. Patients were prepared for Galdakao-modified supine Valdivia (GMSV) position [16] (Figure 2). Two groups of doctors were needed. MPCNL group was doing second stage MPCNL using the former single-tract access. Briefly, a 0.035 guidewire is inserted into the renal calyx though the nephrostomy tube. Tract dilatation was serially accomplished using Amplatz dilators up to the match size, and working sheath was advanced into the renal collecting system. Nephroscope was inserted into the renal collecting system. Simultaneously, RIRS was performed by another group. (Figure 2). After retrograde pyelography and placing a safety guidewire, a 12/14 ureteric access sheath was advanced into the proximal ureter, allowing easy passage of the 7.5 F flexible ureteroscope into the collecting system. The renal stones and collecting system were carefully inspected. Residual stones in the peripheral calyces, which would have required a second or third nephrostomy access, were fragmented with a 200 µm holmium laser fiber. Alternatively, a stone basket or grasping devices was used to transfer calyceal stones into the renal pelvis, where the MPCNL group can use concomitant lithotripsy and remove the fragments efficiency. At the end of procedure, the collecting system accessible to the rigid and flexible nephroscope was examined for potential fragments. Patients were discharged home 2 days after second stage procedure, following removal of their nephrostomy tube, provided no significant residual stone was seen on the KUB.

### Follow-up

One month after the final procedure, all patients were assessed by CT to confirm the final SFR. Complete stone-free was defined as the absence of any fragments in kidney or had clinically insignificant residual fragments (CIRFs), defined as 4 mm or smaller, nonsymptomatic, nonobstructive and noninfectious residual fragments [17]. The Double-J stent would be removed in the complete stone-free patients.

After the first follow-up evaluation, patients returned for an assessment with urinalysis, serum creatinine, KUB and urinary ultrasound was used every 3 months during the first year and every 6 months thereafter. When stones appeared in stone-free patients or the size of residual stones increased, patients were reevaluated by CT to guide the appropriate treatment modality.

Statistical analysis was done with SPSS 16.0® for Windows®. Continuous variables were compared with student t test and Wilcoxon test, and Proportions of categorical variables were analyzed for statistical significance using the Fisher exact test. Differences resulting in p<0.05 were considered significant.

## Results

The study identified 20 patients, including 8(40%) men and 12(60%) women. Mean age was 52.75±13.13 years (range 21 to 77) and mean BMI was 22.98±3.51 kg/m^2^ (range 17.76 to 31.99). Mean stone burden was 1099.9±843.95 mm^2^ (range 291 to 2925).Five patients had a previous renal intervention history in the procedure side. Six staghorn calculi were in the right side, and 14 were in the left. All patients were suffered from medical comorbidities (1 cardiac; 2 diabetes; 5 hypertension; 12 renal failure).15(75%) patients were identified as hydronephrosis, including 8(40%) mild, 4(20%) moderate and 3(15%) severe. Preoperative urine cultures were positive in 10 (50%) patients. All these infections were administrated with culture-specific antibiotics. Demographic and clinical preoperative characteristics of patients were shown in Table 1. During the preoperative period, one (5%) patient had CKD stage 1, four (20%) had stage 2, eight (40%) had stage 3, five (25%) had stage 4 and two (10%) had stage 5. Two patients required dialysis preoperatively.

**Table 1:** Demographic and clinical preoperative characteristics of patients.

Age, year, mean(SD), range	52.75(13.13), 21–77
Sex, no.(%)	
Male	8(40)
Female	12(60)
BMI, kg/m^2^, mean (SD)	22.98(3.51)
Cause of solitary kidney, no. (%)	
Congential	3(15)
Previous nephrectomy	10(50)
Nonperfused kidney	7(35)
Stone side, no. (%)	
Left	14(70)
Right	6(30)
Staghorn Stone type, no. (%)	
Complete	7(35)
Partial	13(65)
Stone burden, mean (SD), range (mm^2^)	1099.9(843.95), 291–2925
Previous renal intervention history,no, (%)	
Open surgery	2(10)
ESWL	2(10)
PCNL	1(5)
Grade of hydronephrosis, no. (%)	
None	5(25)
Mild	8(40)
Moderate	4(20)
Severe	3(15)
Medical Comorbidities, no. (%)	
Hypertension	5(25)
Diabetes	2(10)
Cardiac	1(5)
Renal failure	12(60)
Positive preoperative urine culture,no. (%)	10(50)
E. coli	3(15)
Enterococcus faecalis	1(5)
Acinetobacter	1(5)
Pseudomonas aeruginosa	1(5)
Enterobacter cloacae	2(10)
Proteus mirabilis	2(10)

All patients had only one percutaneous access tract. Access to calices through an 11th intercostal route was established in 17 renal units, and sub-12th costal approach was in three renal units. The mean operative duration was 96.88±30.42 min in one stage and 87.86±34.90 min in second stage. The mean whole operative duration was 154.37±32.45 min, which was not significantly different from previous experience with standard multiple accesses PCNL had reported [18], [19]. The mean blood loss was 64 (12–140) ml, which was less than multiple-puncture PCNL had reported [18], [19]. There was no intraoperative complications in all cases. Postoperative complications noted in 5(25%) patents according to the Clavien classification. Four patients had fever on the day 1 after MPCNL, which resolved spontaneously in two, while the remaining two patients required antibiotic treatment because of positive urine cultures suggested urinary tract infection, and positive blood cultures. Two patients were treated with a complete culture-specific antibiotics until body temperature, urinalysis and urine culture were normal. One patient was prolonged tract leakage because of diabetes. No patients required blood transfusion. Mean hospitalization time was 12.11±2.77 days, two patients prolonged hospitalization time because of poor wound healing induced by diabetes. The Initial SFR after the procedure was 95%. One patient had small asymptomatic residual calculi (largest 6 mm) in the lower pole. Three patients had CIRFs. The final SFR was 90%, confirmed by CT 1 month after the final operation. Stone recurred in one patient in the lower pole (diameter<6 mm, asymptomatic). Operative characteristics and stone composition were shown in Table 2. During the whole follow-up period, another patient had recurred stone (diameter<12 mm, in the upper pole) in postoperative 6 months, who was treated with RIRS. Stone recurred was not found in the rest patients.

CKD stage was compared in Table 3 before and after the treatment. During the 1-month follow-up study period, 4 patients improved to be CKD (stage 3) postoperatively, who were identified as CKD (stage 4) preoperatively. Two patients who had CKD (stage 5) still needed dialysis postoperatively. No more patients progressed to end-stage renal disease requiring dialysis. Mean Scr of the rest patients before operation was 187.16±94.12 umol/L compared to 140.99±57.92 umol/L by the end of 1-month follow-up period with statistical significance (p = 0.019). The same findings were observed in GFR in that before operation it was 43.80±24.74 ml/min and by the end of the 1-month follow-up it was 49.55±21.18 ml/min with statistical significance (p = 0.05). However, there were no statistically significant differences between these parameters from 1 month to 1-year follow-up (p>0.05). Table 3 also compared Scr and calculated GFR of 18 patients before and after the treatment.

**Table 2:** operative characteristics and stone composition of patients.

Puncture (one staged MPCNL), no. (%)	
Posterior middle calyx via 11^th^ intercostal	17(85)
Posterior lower pole below 12^th^ costal	3(15)
No. of tracts, no. (%)	
Single	20(100)
Access tract Size, no. (%)	
18 F	17(85)
20 F	3(15)
Time to access the collecting system, mean, (SD), range (min)	14.3(11–23)
Residual calculi characteristics after one staged MPCNL	
Stone size, mean, (SD), range (mm^2^)	743.12(312.34), 112–1831
Stone position, no. (%)	
Upper pole	6(30)
Middle calyx	2(10)
Lower pole	1(5)
Mix	11(55)
Operative duration, mean (SD), range(min)	
One stage	96.88(30.42), 65–175
Second stage	87.86(34.90), 30–165
The whole	154.37(32.45), 99–224
Mean blood loss, mean (SD), range(ml)	64(13.2), 12–140
Stone-free rate, no. (%)	
Initial SFR	19(95%), lower pole residual small fragments largest 6 mm diameter.
Final SFR	18(90%), two patients with largest 6 mm fragments in lower pole.
Nephrostomy removal time, Mean, (SD), range (days)	6.3(0.5), 5–11
Hospitalization time, Mean, (SD), range (days)	12.11(2.77), 8–22
Lithotripsy, no. (%)	
Holmium laser	20(100)
Pneumatic lithotripter	14(70)
Cyberwand® dual probe ultrasonic	10(50)
Complications of operation, no. (%)	
Postoperative fever (>38.5°C)	4(20)
Prolonged tract leakage	1(5)
Urinary tract infection	2(10)
Stone analysis	
Calcium oxalate	5(25)
Calcium oxalate and phosphate	6(30)
Uric acid	2(10)
Struvite	3(15)
Mix	4(20)

## Discussion

In patients with solitary kidney, an untreated staghorn calculus is likely to destroy the renal function and cause life-threatening urosepsis. Complete removal of the stone is crucial aim to eradicate any causative organisms, relieve obstruction, prevent further stone recurrence and preserve the renal function as far as possible [20]. PCNL is currently the recommended treatment for patients with staghorn calculi based on the superior outcomes and acceptable low morbidity, with the advent of endourologic instruments, lithotripsy devices, and surgical techniques [1], [21]. Although rare, PCNL is associated with significant complications, especially in patients with solitary kidney. One of the most common and worrisome complications following PCNL is renal hemorrhage [22]. Because of physiological compensatory hypertrophy of the renal parenchyma in the solitary kidney, it is more likely to increase the risk of hemorrhage owe to damaging more renal tissue and blood vessel in puncturing and dilating the thick renal parenchyma [23]. Previous study has demonstrated that compared with the tract of standard PCNL (26–30 F), the small tract (12–20 F) of MPCNL obviously reduced the damage of renal parenchyma and vessel. Although, previous literature has proved that mean intrapelvic pressures of the minimum tract (14 F) in MPCNL was still lower than the causing backflow (30 mmHg) [24], performing PCNL in lower intrapelvic pressures is effective to reduce the absorption of toxinum and pyrogen. In this study, 17(85%) MPCNL was performed with 20 F because each effective lithotripsy can be placed through the nephroscope in this size of working sheath. Accessing the collecting system through the posterior middle calyx provided the most direct and shortest tract from skin to renal collecting system, and may enable to access the majority of the collecting system. Performing MPCNL by an 8/9.8 F rigid ureteroscope was usual in the past, with the advantage of its slender body, which can access to more calyces to acquire a higher SRF and a lower complication in a single tract [8], [25]. However, urologists often complained of fatigue, bad visual fields, and low efficiency in stone removal, when using ureteroscope. With the development of endourology, an 8.5/12 F rigid nephroscope was designed to overcome the disadvantage of ureteroscope in MPCNL procedure. It not only has a relative suitable body (12 F), which can access to most calyces, but also had a wider working channel (8.5 F), which is to facilitate stone removal with a forceps. Addition, because front mouth of the nephroscope is flat, the small fragments were pushed out with an endoscopic pulsed perfusion pump and suck out with suction easily. The valves of the nephroscope were connected with pulsed perfusion pump and suction, which can be regulated by the urologist. This convenient regulated system was used so that irrigation fluid could escape easily with no increase in the intra-pelvic pressure. An 18 F rigid nephroscope was designed specially for dual probe ultrasonic intracorporeal lithotripter, which was a high effective lithotripter in managing large bulk stone with low intra-pelvic pressure through a single tract.

**Table 3:** Serum creatinine, GFR of 18 patients and CKD stage of all patients before and after operation.

	Preoperative	Postoperative,1 month	p value
Serum creatinine(umol/L)			
Mean(SD)	187.16(94.12)	140.99(57.92)	0.019
Range	77–580	77–420	
GFR (ml/min)			
Mean (SD)	43.80(24.74)	49.55(21.18)	0.05
Range	15.12–104.36	22.46–104.36	
CKD stage, no. (%)			0.48
1	1(5)	1(5)	
2	4(20)	4(20)	
3	8(40)	12(60)	
4	5(25)	1(5)	
5	2(10)	2(10)	

Access to all the calyces through one percutaneous tract may be difficult due to the peculiar anatomical structure of the collecting system. Meanwhile, solitary kidney with large burden stone, branched stone and satellite stone or residual stone in parallel calyces of the access often requires multiple access tracts during PCNL. Furthermore, multiple access tracts may be associated with greater risk of renal hemorrhage [26], [27]. RIRS has been used successfully to access and treat complex renal calculi smaller than 2 cm with high reported stone-free rates [9]. RIRS has fewer overall complications compared to PCNL, even absence of renal injure. However, the small working channels of RIRS had limited the usefulness of effective instrumentation that allows concurrent stone fragmentation and removal [18]. Multiple procedures or combined approach may be required to clear a large stone. Although, Wong et al [28] in their study could achieve clearance rates of 95% in 45 renal units with a single puncture followed by flexible nephroscopy in a staged manner, SRF after the first stage was only 51.4%, and flexible nephroscopy with a large diameter was not easy to access to the narrow calyces. Perhaps, the same group of patients would have been rendered completely stone free with the addition of multiple tracts even at the cost of extending the hospital stay. Landman et al [29] use simultaneously ureteroscopy and lower calix single-tract PCNL for single-access of six complete and three partial staghorn calculi with no major complication. But SRF was only 78%. Marguet et al [18] applied the same concept, albeit with the difference that ureterorenoscopy was performed firstly to clear the stones in the peripheral calices, which would have needed a second or third nephrostomy access. Then the patient was placed in the prone position and single access PCNL performed to remove the residual stone.

In this study, combination of single-tract MPCNL and RIRS was performed in the second stage, when the drainage was clear and the visual fields were well. Take the advantage of single tract one-stage MPCNL, the residual stone burden can be fragmented by holmium laser or translocated into the renal pelvis, using a basket or grasp by RIRS doctor group. Simultaneously, MPCNL doctor group can manage the stone with effective intracorporeal lithotripsy or removed them with a forceps easier for previous access. Despite retrograde irrigation, stone fragments often fail to clear completely after intracorporeal lithotripsy. Combination of single-tract MPCNL and RIRS offers a larger bore access that can allow efficient removal of these remaining fragments. The mean whole operative duration was not significantly different from previous experience with standard multiple accesses PCNL had reported. The mean blood loss was 64 (12–140) ml, which was less than multiple-puncture PCNL had reported by other urologist [18], [19]. The initial and final SFR were 95% and 90%, which was higher than the PCNL monotherapy (74% - 83%) [4] and Landman’s study [29].

Previous study has demonstrated that no significant change in estimated Scr and GFR under PCNL with solitary kidney [28], [30]. However there are at present no available well-constructed trials comparing the long-term renal effects of multiple-tract PCNL with single-tract PCNL. According to our surgical approach, regardless of 2 dialysis patients, a significant improvement in Scr and GFR was demonstrated. Mean Scr before operation was 187.16±94.12 umol/L compared to 140.99±57.92 umol/L by the end of 1-month follow-up period with statistical significance (p = 0.019). The same findings were observed in GFR in that before operation it was 43.80±24.74 ml/min and by the end of the 1-month follow-up it was 49.55±21.18 ml/min with statistical significance (p = 0.05). Four patients improved by CKD stage. However, there were no statistically significant differences between these parameters from 1 month to 1-year follow-up (p>0.05). As is known, the most deleterious effect on renal function is neglected obstruction. If obstruction in a solitary kidney is rapidly relieved the kidney will regain basal function. It seems that our surgical approach did not adversely affect renal function.

In conclusion, our surgical approach modified here effectively decreases the number and size of percutaneous access tracts, which combines the advantages of both MPCNL and RIRS. It is safe, feasible, and efficient for managing staghorn calculi in solitary kidney with satisfactory SFR and reducing blood loss, potential morbidity associated with multiple tracts. The approach did not adversely affect renal function at both short-term and long-term follow-up.

### Statement

The subjects included in figure 2 have given written informed consent, as outlined in the PLoS consent form, to publication of their photograph.
